# Significance of AtMTM1 and AtMTM2 for Mitochondrial MnSOD Activation in *Arabidopsis*

**DOI:** 10.3389/fpls.2021.690064

**Published:** 2021-08-06

**Authors:** Shu-Hsuan Hu, Shu-Fan Lin, Ya-Chen Huang, Chien-Hsun Huang, Wen-Yu Kuo, Tsung-Luo Jinn

**Affiliations:** ^1^Institute of Plant Biology and Department of Life Science, National Taiwan University, Taipei, Taiwan; ^2^Institute of Plant Biology, Center of Evolutionary Biology, School of Life Sciences, Fudan University, Shanghai, China

**Keywords:** metalloenzyme, mitochondrial carrier family, MnSOD, Mn transporter, reactive oxygen species, superoxide dismutase

## Abstract

The manganese (Mn) tracking factor for mitochondrial Mn superoxide dismutase (MnSOD) has been annotated as yMTM1 in yeast, which belongs to the mitochondrial carrier family. We confirmed that *Arabidopsis* AtMTM1 and AtMTM2 are functional homologs of yMTM1 as they can revive yeast MnSOD activity in *yMTM1*-mutant cells. Transient expression of AtMnSOD-3xFLAG in the *AtMTM1* and *AtMTM2*-double mutant protoplasts confirmed that AtMTM1 and AtMTM2 are required for AtMnSOD activation. Our study revealed that AtMnSOD interacts with AtMTM1 and AtMTM2 in the mitochondria. The expression levels of *AtMTM1*, *AtMTM2*, and *AtMnSOD* respond positively to methyl viologen (MV) and metal stress. AtMTM1 and AtMTM2 are involved in Mn and Fe homeostasis, root length, and flowering time. Transient expression of chloroplast-destined AtMnSOD revealed that an evolutionarily conserved activation mechanism, like the chloroplastic-localized MnSOD in some algae, still exists in *Arabidopsis* chloroplasts. This study strengthens the proposition that AtMTM1 and AtMTM2 participate in the AtMnSOD activation and ion homeostasis.

## Introduction

Cellular reactive oxygen species (ROS) contain superoxide anion radicals, hydroxyl radicals, singlet oxygen, and hydrogen peroxide, whose generation is induced by high light intensity, heat, drought, and salt stress. Superoxide anion radicals are mainly generated from the respiratory and photosynthetic electron transport chains in the mitochondria and chloroplasts and can rapidly damage nearby cell components. The superoxide dismutases (SODs) catalyze the conversion of toxic superoxide anion radicals to oxygen and hydrogen peroxide; the corresponding cofactors in SODs are transition metal ions that accept or donate an electron during the dismutation process (Fridovich, 1975; Halliwell, 1994; Apel and Hirt, 2004).

Superoxide dismutases are classified as CuZnSOD, FeSOD, Mn superoxide dismutase (MnSOD), or NiSOD based on their metal cofactors. These metalloenzymes are important for cell survival under oxidative stress (Bowler et al., 1994). Most eukaryotes contain CuZnSOD and MnSOD, while plants also contain FeSOD (Alscher et al., 2002; Fink and Scandalios, 2002). NiSOD is present in *Streptomyces* and cyanobacteria (Choudhury et al., 1999; Barondeau et al., 2004; Priya et al., 2007). In *Arabidopsis* (*Arabidopsis thaliana*), seven SOD isoforms are distributed among various organelles, including cytosolic CuZnSOD1, chloroplastic CuZnSOD2, and peroxisomal CnZnSOD3, as well as three chloroplastic FeSODs and one mitochondrial MnSOD (Kliebenstein et al., 1998; Kuo et al., 2013a,b). MnSOD is also found in the thylakoid membrane of some species of green and blue–green algae.

The SOD enzyme activation requires a metallochaperone or transporter that captures and loads the metal cofactor into the SOD apoprotein. The pathway using copper chaperone of SOD1 (CCS) for CuZnSOD activation is referred to as the CCS-dependent pathway (Casareno et al., 1998; Rae et al., 2001; Chu et al., 2005; Culotta et al., 2006). The chloroplast chaperonin Cpn20 functions as an Fe chaperone for FeSOD activation (Kuo et al., 2013a,b). The manganese (Mn) tracking factor for mitochondrial MnSOD activation was annotated as yMTM1 in yeast; its homolog AtMTM1 can complement yeast MnSOD (ySOD2) activity in *yMTM1* mutant cells (Luk et al., 2003; Su et al., 2007). Notably, Mn supply increased the ySOD2 activity in yeast cells, and the ySOD2 protein could not be activated in the cytosol, suggesting that Mn insertion is linked to the ySOD2 importing process via yMTM1 in mitochondria (Luk et al., 2005).

There are 35 members of the mitochondrial carrier family in yeast, and more than 50 members in plants and humans, which are evolutionarily conserved for transporting cofactors and specific substrates (Haferkamp and Schmitz-Esser, 2012). *Arabidopsis* AtMTM1 and AtMTM2 are classified as mitochondrial carriers with conserved transmembrane sequences, and their amino acid sequences are highly homologous in the phylogeny (Picault et al., 2004; Palmieri et al., 2011). To date, the physiological roles of AtMTM1 and AtMTM2 in MnSOD activation have not been fully elucidated.

This study revealed that the effects of AtMTM1 and AtMTM2 on MnSOD activation in *yMTM1* mutant cells and *AtMTM1* and *AtMTM2*-double mutant plants are similar. We confirmed that AtMTM1 and AtMTM2 are associated with root length and flowering time. We also found that AtMTM1 and AtMTM2 have different responses under stress, and both play a role in Mn homeostasis. Finally, our study revealed that the activation mechanism of the chloroplastic-localized MnSOD in algae is retained in *Arabidopsis* chloroplasts.

## Materials and Methods

### Plants and Growth Condition

*Arabidopsis* (*A. thaliana*) accession Columbia-0 (Col) was used as the wild-type (WT). T-DNA insertion mutants of *mtm1-1* (SALK 023286), *mtm1-2* (SALK 054287C), *mtm2-1* (SALK 005166), *mtm2-2* (SALK 103984), and *mtm2-3* (SALK 025071) were obtained from the Arabidopsis Biological Resource Center (ABRC, Ohio State University, Columbus, United States). Plants were grown in growth chambers at 22–24°C with 8 h dark/16 h light at 80–100 μmol m^–2^ s^–1^. For root length measurements, sterile seeds were placed on solid half-strength Murashige and Skoog (1962) basal medium (1/2 MS; Sigma M5519) containing 1% sucrose and 0.8% (w/v) phytagel (Sigma, Ronkonkoma, NY, USA). For gene regulation, metal stress, and metal ion homeostasis, 14-day-old complete seedlings were transferred to Milli-Q water containing methyl viologen (MV; paraquat), metals, or MnCl_2_. Transgenic plants were generated by *Agrobacterium tumefaciens* GV3101-mediated transformation and the floral dip method (Clough and Bent, 1998). The flowering time was scored when the primary inflorescence reached 5 cm in length.

### Yeast Strains and Growth Condition

Yeast (*Saccharomyces cerevisiae*) used in this study contained WT BY4741 (Y00000; *MATa*, *his3*Δ*1*, *leu2*Δ*0*, *met15*Δ*0*, and *ura3*Δ*0*) and BY4742 (Y10000; *MAT*α, *his3*Δ*1, leu2*Δ*0, lys2*Δ*0*, and *ura3*Δ*0*). The mutant strains of *ysod2*Δ (Y06605; *sod2:kanMX4*) and *ymtm1*Δ (Y07288; *ygr257c:kanMX4*) were obtained from the Saccharomyces Genome Deletion Project^1^. Enriched yeast extract–peptone–dextrose (YPD) medium was supplemented with 2% glucose to culture yeast at 30°C under aerobic conditions (Brachmann et al., 1998). For yeast expression, all cDNAs were cloned into the yeast expression vector pADNS (Colicelli et al., 1989). Yeast transformation was performed according to the lithium acetate procedure (Gietz and Schiestl, 1991), and transformants were selected on minimal synthetic dextrose (SD) media.

### Western Blotting and SOD Activity Analysis

Yeast lysate was extracted by using the glass bead lysis method (Culotta et al., 1997). Total protein from *Arabidopsis* seedling was isolated by grinding 100 mg of frozen tissue in 300 μl of ice-cold buffer of 50 mM potassium phosphate (pH 7.8), 0.1% BSA, 0.1% ascorbate, and 0.05% 2-mercaptoethanol (Van Camp et al., 1994), as well as in 150 mM Tris–HCl buffer (pH 7.2) (Pan and Yau, 1992; Chen and Pan, 1996; Chu et al., 2005; Kuo et al., 2013c). Supernatants were collected by centrifuging twice for 10 min at 16,000 × *g* at 4°C in Eppendorf tube, and protein concentration in the supernatants was determined using the Bradford protein assay method (Bradford, 1976). An equal amount of proteins were separated immediately on a 10% non-denaturing polyacrylamide gel for in-gel SOD activity assay (Beauchamp and Fridovich, 1971; Kliebenstein et al., 1998; Kuo et al., 2013c). A 12.5% denaturing polyacrylamide gel electrophoresis was used for western blotting with antibodies of α-ADH1 (Sigma-Aldrich, St. Louis, MO, USA), α-FLAG (Sigma-Aldrich), α-Actin (Agrisera), and α-AtMnSOD (Agrisera, Västerbäck, Vännäs, Sweden). The SOD activity was quantified by the UVP ChemStudio PLUS imaging system (Analytik Jena US LLC, Upland, CA, United States).

### Genomic DNA and RNA Preparations, cDNA Synthesis, and Quantitative Real-Time PCR

Genomic DNA was extracted for genotyping using PCR, as previously reported (Edwards et al., 1991). Total RNA was prepared with TRIZOL reagent (Invitrogen, Carlsbad, CA, USA) and TURBO DNA-free Kit (Applied Biosystems, Foster City, CA, USA). cDNA synthesis was performed with Ready-To-Go Kit (GE Healthcare Life Sciences, Stockholm, Sweden) and analyzed by reverse transcription PCR (RT-PCR). PCR primers were designed by Primer3^2^. Quantitative real-time PCRs (qPCRs) with iQ SYBR Green Supermix (Bio-Rad, Techview, Singapore) were performed by using MyiQ thermocycler and iQ5 optical system (Bio-Rad). qPCR data were normalized to the internal control of *PP2AA3* (*PP2A*) (Czechowski et al., 2005).

### Subcellular Localization and Bimolecular Fluorescence Complementation

The coding regions of *AtMTM1*, *AtMTM2*, and *AtMnSOD* genes were amplified by RT-PCR, and cloned into pCR8/GW/TOPO vector (Invitrogen) for sequencing. Constructs were further recombined into pEarleyGate101 vector that contains yellow fluorescent protein (YFP) for subcellular localization or into pEarleyGate201-YN and pEarleyGate202-YC for bimolecular fluorescence complementation (BiFC) assay (Lu et al., 2010). Four-week-old plants were used for protoplast preparation and transfection (Yoo et al., 2007). Transient expression of 15 μg plasmid DNA in 2 × 10^4^ protoplasts was performed in BiFC assay, and YFP signals were observed by using a TCS SP5 confocal microscope (Leica, Wetzlar, German). Mitochondria were stained with 200 nM MitoTracker CMTMRos (Thermo Fisher Scientific, Fremont, CA, USA). The fluorescence signals of YFP, MitoTracker, and chlorophyll were all excited at 514 nm and recorded at 530, 580, and 660 nm, respectively.

### Generation of *AtMTM1*-miRNA Mutants, Complementation Lines With *AtMTM2*, and *AtMTM2* Histochemical Analysis

The 21mer artificial miRNA of *AtMTM1* was cloned into the pRS300 vector which contains the miR319a precursor (Schwab et al., 2006). The artificial miRNA^3^ was designed and cloned into pPZP200GB vector (Chu et al., 2005) for constructing miRNA-mediated *AtMTM1* suppression (*mtm1-i*) lines. The construct of *35S:AtMTM2* with pCAMBIA1300 vector (CAMBIA) was transformed into *mtm1-i*, *mtm2*, and *mtm1-i mtm2*-double mutants background. These complementation lines with *AtMTM2* transgene were selected by hygromycin and kanamycin. In addition, a 2-kb *AtMTM2* promoter region was subcloned into pCAMBIA3300 vector (CAMBIA) with a GUS reporter for analyzing the *AtMTM2* promoter activity. The GUS staining method was performed as described (Weigel and Glazebrook, 2002).

### Inductively Coupled Plasma Optical Emission Spectrometry

Inductively coupled plasma optical emission spectrometry (ICP-OES) (Perkin Elmer Optima 5300 DV) was conducted to evaluate the metal ion content (Lahner et al., 2003; Yang et al., 2008; Lin et al., 2009); 14-day-old complete seedlings were transferred to the 1/2 MS medium containing 100 μM MnCl_2_ for 24 h with agitation. Aerial parts (shoots) and roots were separated and dried at 60°C for 3 days. An amount of 0.1 g dried tissue sample was analyzed by using the ICP-OES. Spinach and tomato leaves (SRM-1570a and SRM-1573a; US National Institute of Standards and Technology) were the standard references to validate the measurement.

### Constructs of Different Organelle-Destined AtMnSOD

The cytosol-destined and chloroplast-destined AtMnSOD constructs were generated (Supplementary Figure 1A). The deletion of the mitochondrial transit peptide of AtMnSOD resulted in cytosol-localized AtMnSOD (Δ-TP-AtMnSOD). The mitochondrial transit peptide of AtMnSOD replaced by the chloroplast transit peptide of *Arabidopsis* CuZnSOD2 (Huang et al., 2012) became chloroplast-destined AtMnSOD (Chl-TP-AtMnSOD). They were further recombined into the pEarleyGate101 vector for confirmation of subcellular localization. Besides, AtMnSOD-Tag (a tag of 15 amino acids derived from the vector) and AtMnSOD-3xFLAG were generated (Supplementary Figures 1B,C) in pEarleyGate101 vector for transient expression assay in protoplasts.

### Statistical Analysis

All experiments were repeated independently at least three times. Statistical analysis involved Student’s *t*-test and ANOVA with Tukey’s HSD *post hoc* test. *P* < 0.05 was considered statistically significant.

### Primers and Accession Numbers

Primers and accession numbers are listed in Supplementary Table 1.

## Results

### Protein Structure of AtMTM1, AtMTM2, and yMTM1

**FIGURE 1:**
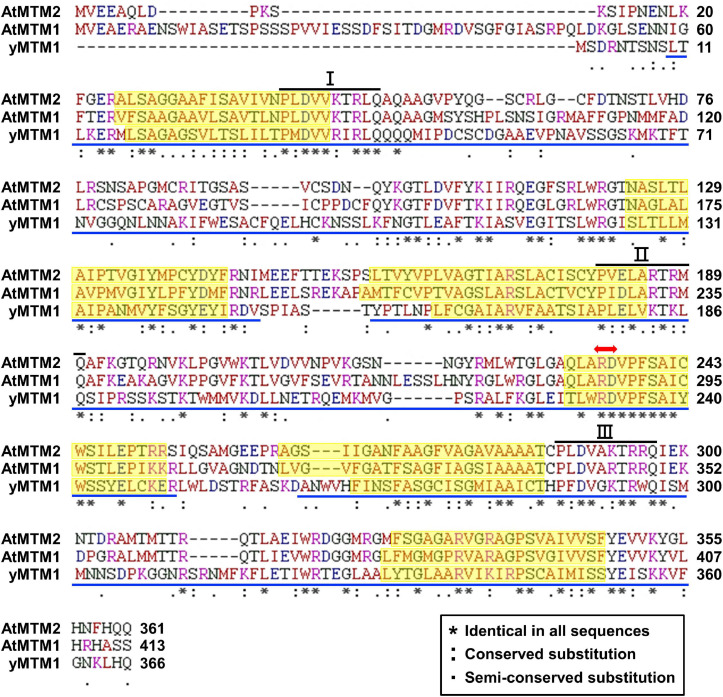
Sequence alignment of AtMTM1, AtMTM2, and yMTM1. Tandemly repeated domains (blue underline), transmembrane α-helices (yellow shading), mitochondrial energy transfer signature (I, II, and III), and substrate Mn-binding site (red double-headed arrow) are indicated. Consensus symbols of identical, conserved, and semi-conserved residues are shown in inset.

We characterized AtMTM1 and AtMTM2 by sequence alignment with reported yMTM1 (Luk et al., 2003; Su et al., 2007), and the results showed that they have high sequence similarity (Figure 1). Each protein sequence consists of three tandem homologous domains, and each domain contains two transmembrane α-helices. Three copies of a 10-amino acid sequence motif are the mitochondrial energy transfer signatures of the mitochondrial carrier family (Millar and Heazlewood, 2003; Robinson and Kunji, 2006). AtMTM1 and AtMTM2 share a conserved Mn-binding site with yMTM1. Protein BLAST^4^ revealed that AtMTM2 has 59% protein identity with AtMTM1 and 34% protein identity with yMTM1. Sequence alignment and motif structure indicated that AtMTM1 and AtMTM2 could be homologs of yMTM1.

### Characterization of *AtMTM1*, *AtMTM2*, and *AtMTM1 AtMTM2*-Double Mutant Lines

To elucidate the physiological functions of *AtMTM1* and *AtMTM2*, we characterized T-DNA insertion mutants of *mtm1-1* and *mtm1-2* and established microRNA-mediated *AtMTM1* mutant (*mtm1-i*) lines. Homozygous T-DNA insertion mutants of *mtm2-1*, *mtm2-2*, and *mtm2-3* were also analyzed.

In *mtm1-1* and *mtm1-2* mutants, T-DNA was inserted in the 3′-UTR and 5′-UTR of the *AtMTM1* gene, respectively (Supplementary Figure 2). Homozygous lines were confirmed by PCR-based genotyping. RT-PCR and qPCR revealed that *mtm1-1* and *mtm1-2* are knockdown mutants. Moreover, five independent T4 lines of the *mtm1-i* mutant were screened (Supplementary Figure 3). The lowest expression line retained approximately 20% of the *AtMTM1* expression and was referred to as *mtm1-i*; however, AtMnSOD activity in *mtm1-i* remained the same as in the WT.

In the *mtm2-1* mutant, T-DNA was inserted in the last exon of the *AtMTM2* coding region (Supplementary Figure 4). Genotyping, RT-PCR, and qPCR confirmed that *mtm2-1* is a null mutant. AtMnSOD activity in *mtm2-1* was also similar to that in the WT. In *mtm2-2* and *mtm2-3* mutants, T-DNA was inserted in the intron, but *AtMTM2* expression was not significantly affected. In the following part of the study, *mtm2-1* refers to the null mutant *mtm2*.

To determine the synergistic effect of *AtMTM1* and *AtMTM2*, we crossed *mtm1-i* and *mtm2* to generate *mtm1-i mtm2*-double mutants and screened the lowest gene expression line (Supplementary Figure 5). Three individual *mtm1-i mtm2*-double mutants exhibited 70% AtMnSOD activity and early flowering phenotype.

### Functional Homologs of AtMTM1, AtMTM2, and yMTM1 in MnSOD Activation

**FIGURE 2:**
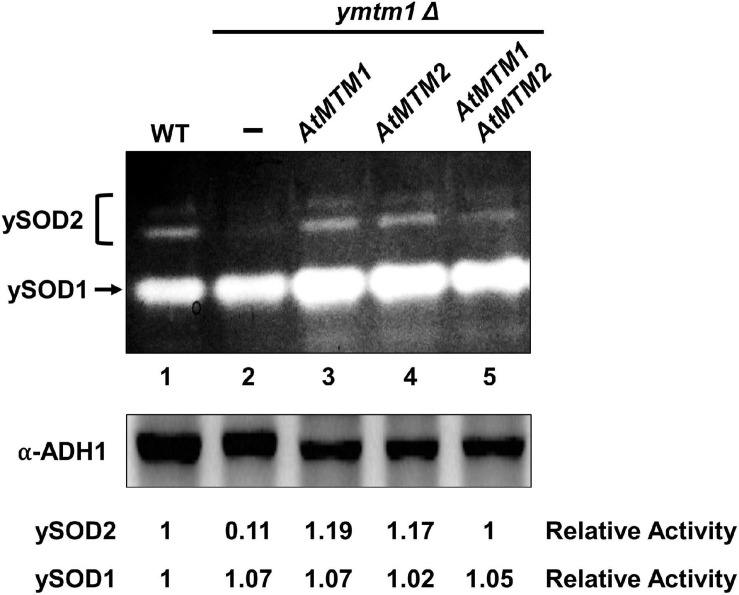
Restored yeast MnSOD activities by AtMTM1 and ATMTM2 in *ymtm1*Δ cells. *ymtm1*Δ cells were transformed without (–) and with *AtMTM1*, *AtMTM2*, and both. Yeast MnSOD (ySOD2) and CuZnSOD (ySOD1) activities are indicated by bracket and arrow, respectively **(top)**. Western blotting with α-alcohol dehydrogenase1 (ADH1) antibody was a loading control **(bottom)**. Relative activity represents ratio of ySOD2 or ySOD1 activity to that in WT (lane 1).

To analyze the role of AtMTM1 and AtMTM2 in yeast MnSOD (ySOD2) activation, we transformed *AtMTM1* and *AtMTM2* transgenes into *ymtm1*Δ cells (Figure 2). WT yeast cells contained two activity bands of ySOD2 and one band of CuZnSOD (ySOD1), with the residual ySOD2 activity retained in *ymtm1*Δ cells (Figure 2, lanes 1 and 2). The activity of ySOD2 was restored by the transformation of *AtMTM1* and *AtMTM2* transgenes in *ymtm1*Δ cells (Figure 2, lanes 3 and 4). Co-transformation of *AtMTM1* and *AtMTM2* restored the ySOD2 activity similar to that of the WT (Figure 2, lane 5). Thus, AtMTM1 and AtMTM2 are functional homologs of yMTM1.

**FIGURE 3:**
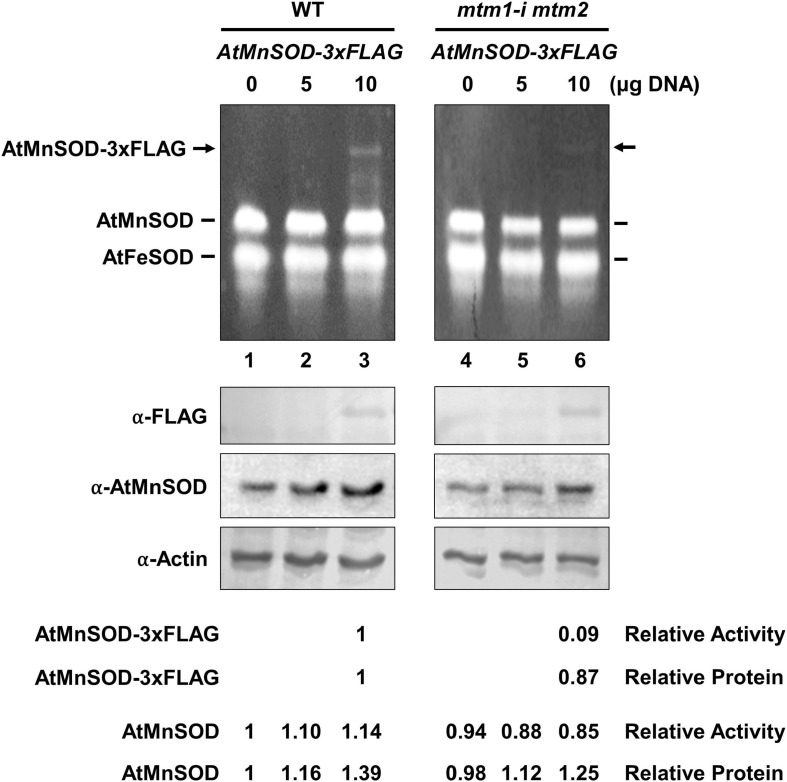
Transient expression of *AtMnSOD-3xFLAG* transgene in WT and *mtm1-i mtm2*-double mutant protoplasts; 5 and 10 μg plasmid DNA containing *AtMnSOD-3xFLAG* transgene were transfected in 10^6^ protoplasts and monitored for 16 h. In-gel SOD activity assay **(top)** and western blotting with α-FLAG, α-AtMnSOD, and α-actin antibodies **(bottom)** were conducted. Actin was a loading control. AtMnSOD-3xFLAG activities and proteins in double mutant cells were measured relative to those in WT (lane 3). AtMnSOD activities and proteins were measured relative to those without transfection (lane 1). Data represent one of three independent repeats.

We also performed a transient expression assay to reveal the role of AtMTM1 and AtMTM2 in AtMnSOD activation in plant cells. We transfected the *AtMnSOD-3xFLAG* transgene in *Arabidopsis* WT and *mtm1-i mtm2*-double mutant protoplasts and monitored them for 16 h (Figure 3). Endogenous AtMnSOD and exogenous AtMnSOD-3xFLAG activities can be distinguished using the in-gel SOD activity assay (Figure 3, top). We compared the relative activity ratio (1:0.09) and the relative protein ratio (1:0.87) of transient-expressed AtMnSOD-3xFLAG between WT and double mutant, and the double mutant exhibited about 10-fold reduction in AtMnSOD-3xFLAG activity with the transfection of 10 μg plasmid DNA (Figure 3, bottom). In addition, endogenous AtMnSOD activity was lower in the double mutant than that in the WT. Overall, it was evident that AtMTM1 and AtMTM2 are crucial for the AtMnSOD activation.

### Subcellular Localization of AtMTM1 and AtMTM2 and Interaction With AtMnSOD

**FIGURE 4:**
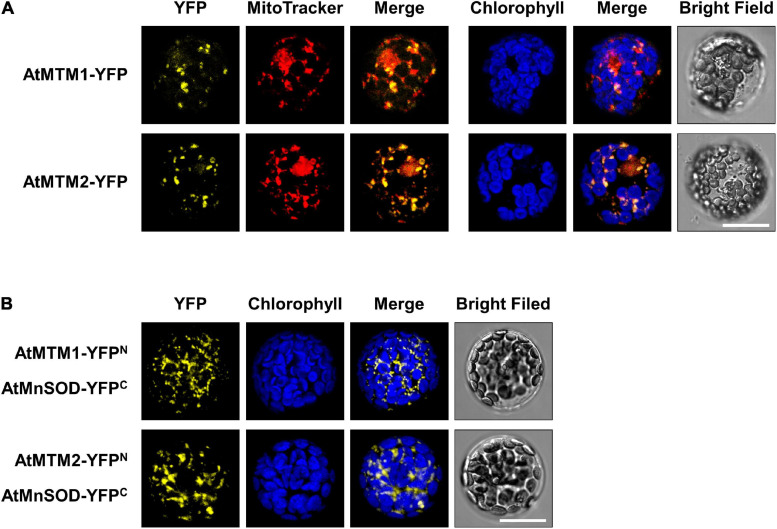
Localization of AtMTM1 and AtMTM2 to mitochondria and their interaction with AtMnSOD. **(A)** *AtMTM1-YFP* and *AtMTM2-YFP* were transfected in *Arabidopsis* protoplasts, and YFP signals were observed by confocal microscopy. **(B)** BiFC assay revealed that AtMnSOD interacted with AtMTM1 and AtMTM2. Reconstituted YFP signals were observed in mitochondria. MitoTracker staining and chlorophyll autofluorescence were used to identify mitochondria and chloroplasts, respectively. Bars = 20 μm.

To examine the subcellular localization of AtMTM1 and AtMTM2, we transfected *AtMTM1-YFP* and *AtMTM2-YFP* transgenes in *Arabidopsis* protoplasts (Figure 4A). The subcellular localization of AtMTM1-YFP and AtMTM2-YFP was examined by using the confocal microscopy analysis. Using the MitoTracker dye, we showed that they merged well with the mitochondria rather than the chloroplasts. This result is in agreement with the proteomic evidence in mitochondrial AtMTM1 and AtMTM2 (Senkler et al., 2017).

A BiFC assay was carried out to examine the protein–protein interactions of AtMnSOD–AtMTM1 and AtMnSOD–AtMTM2 (Figure 4B). The reconstituted YFP signals revealed that AtMnSOD interacts with AtMTM1 and AtMTM2 in the mitochondria. There were no YFP fluorescent signals in the negative controls of the BiFC assay in healthy protoplasts (Supplementary Figure 6).

### Ubiquitous Expressions of *AtMTM1* and *AtMTM2* During Development

The tissue-specific expression of *AtMTM1* was ubiquitous, except in the siliques (Su et al., 2007). The transgenic plants of *AtMTM2*-promoter:*GUS* were established and stained for GUS activity (Supplementary Figure 7). *AtMTM2* expression was clear in the hypocotyls, roots, and cotyledons at an early stage. It was also expressed in the trichomes, vascular bundles, stems, rosettes, and cauline leaves of seedlings. In particular, *AtMTM2* was detected in the floral parts, namely, sepals, petals, anthers, pollen, and stigma, as well as in the young siliques. Overall, *AtMTM2* was ubiquitously expressed during development.

### Expression Levels of *AtMTM1*, *AtMTM2*, and *AtMnSOD* During Development and in Response to Metal Stress

**FIGURE 5:**
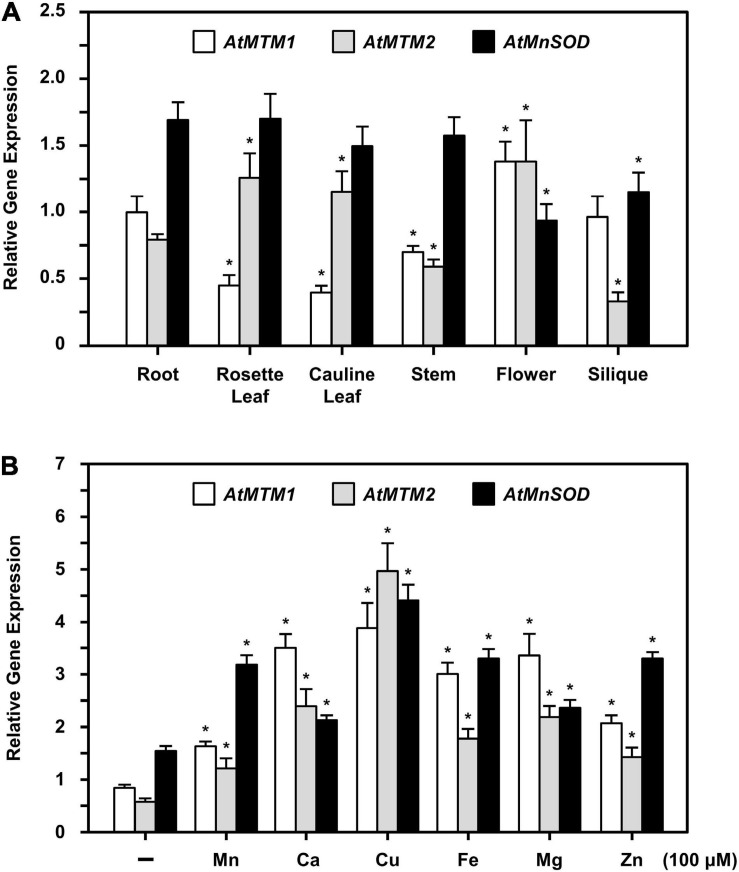
Quantitative PCR analysis of expressions of *AtMTM1*, *AtMTM2*, and *AtMnSOD* in different tissues and under metal stresses. **(A)** Expression levels of *AtMTM1*, *AtMTM2*, and *AtMnSOD* genes were measured relative to *AtMTM1* level in root in WT. **(B)** 14-day-old complete WT seedlings were treated without (–; control) and with metal ions for 24 h. Gene expression levels in response to 100 μM MnCl_2_, CaCl_2_, CuSO_4_, Fe citrate, MgCl_2_, or ZnSO_4_ were measured relative to *AtMTM1* level in control. Data are mean ± SE of three independent repeats. *Significant at *P* < 0.05 (Student’s *t*-test). *PP2A* was an internal control.

The expression levels of *AtMTM1*, *AtMTM2*, and *AtMnSOD* genes in roots, rosette leaves, cauline leaves, stems, flowers, and siliques were examined by qPCR (Figure 5A). *AtMTM1* was higher than *AtMTM2* in the roots, stems, and siliques, while *AtMTM2* was dominant in leaves. Notably, the expression levels of both *AtMTM1* and *AtMTM2* were substantial in flowers. In addition, *AtMnSOD* was consistently higher during development, but was lower in flowers and siliques.

To elucidate the effect of metal stress on *AtMTM1*, *AtMTM2*, and *AtMnSOD* gene regulation, we transferred 14-day-old complete seedlings to Milli-Q water containing 100 μM of Mn (MnCl_2_), Ca (CaCl_2_), Cu (CuSO_4_), Fe (Fe citrate), Mg (MgSO_4_), or Zn (ZnSO_4_) with gentle agitation for 24 h, after which the gene expression corresponding to each metal stress was examined by qPCR (Figure 5B). All three genes were upregulated under metal stress as compared to those under the unstressed condition. In particular, the expression levels of *AtMTM1* were higher than those of *AtMTM2* under all metal stress conditions except Cu stress, implying that *AtMTM1* and *AtMTM2* have different expression sensitivities in response to different metal stresses.

### Divergent Effects of *AtMTM1* and *AtMTM2* in Response to Oxidative Stress

**FIGURE 6:**
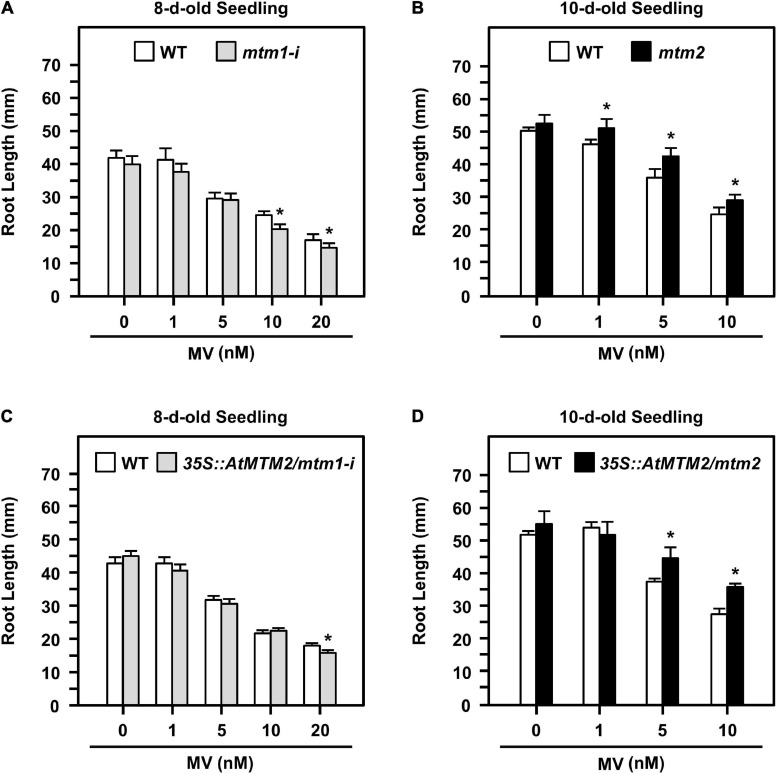
Root lengths of *mtm1-i*, *mtm2*, and complementation lines with *AtMTM2* transgene in *mtm1-i* and *mtm2* backgrounds in response to MV stress. **(A,B)** Root lengths of *mtm1-i* grown on 1–20 nM MV plates on day 8 and *mtm2* grown on 1–10 nM MV plates on day 10, respectively. **(C,D)** Root lengths of complementation lines with *AtMTM2* transgene in *mtm1-i* and *mtm2* backgrounds (*35S:AtMTM2/mtm1-i* and *35S:AtMTM2/mtm2*) on day 8 and day 10, respectively. Data are mean ± SE of three independent repeats (*n* = 50). *Significant at *P* < 0.05 (Student’s *t*-test).

Altered root elongation is an indication of oxidative stress during plant growth (Tsukagoshi, 2012); we used MV as an oxidative stress inducer. We monitored the root lengths of *mtm1-i* and *mtm2* and the complementation lines with the *AtMTM2* transgene in *mtm1-i* and *mtm2* backgrounds (i.e., *35S:AtMTM2/mtm1-i* and *35S:AtMTM2/mtm2*) on MV-containing plates from day 8 to day 10 (Figure 6). The treatment period and MV concentration varied depending on the mutant, and thus, we used 8- to 10-day-old seedlings to compare the complementary effect of *AtMTM2* on mutants with different backgrounds.

The 8-day-old *mtm1-i* seedlings indicated a shorter root length phenotype compared with the WT in response to 10 and 20 nM MV treatments (Figure 6A); however, the 10-day-old *mtm2* seedlings showed longer root length in response to 1, 5, and 10 nM MV treatments (Figure 6B). The complementation lines of *35S:AtMTM2/mtm1-i* recovered root length under 10 nM MV on day 8 (Figure 6C), and the *35S:AtMTM2/mtm2* recovered root length under 1 nM MV on day 10 (Figure 6D). Overall, *AtMTM1* and *AtMTM2* are associated with primary root length control.

### Quick and Positive Response of *AtMnSOD*, *AtMTM1*, and *AtMTM2* to Oxidative Stress

**FIGURE 7:**
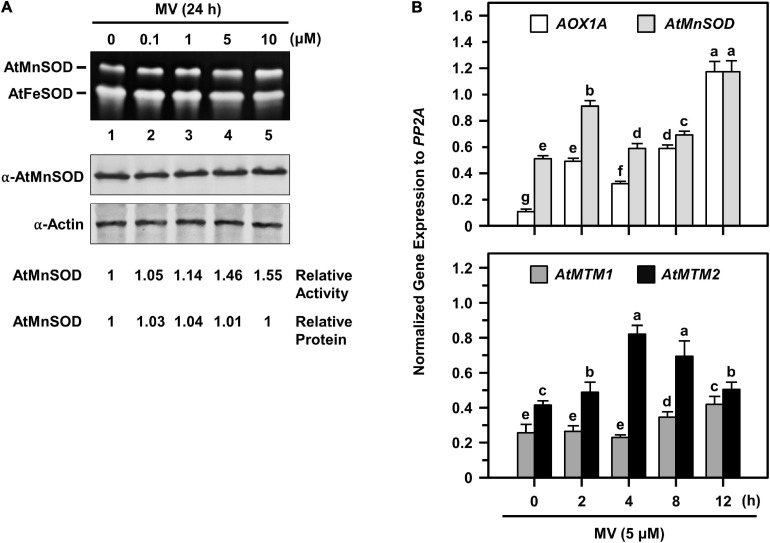
AtMnSOD regulation and gene expressions of *AtMnSOD*, *AtMTM1*, and *AtMTM2* under MV stress. **(A)** 14-day-old complete WT seedlings were treated with 0.1–10 μM MV for 24 h. In-gel SOD activity assay **(top)** and western blotting with α-AtMnSOD and α-actin antibodies **(bottom)** were conducted. Actin was a loading control. AtMnSOD activities and proteins were measured relative to those in control without treatment (lane 1). **(B)** Complete WT seedlings were treated with 5 μM MV for 2–12 h. Expression levels of *AOX1A*, *AtMnSOD*, *AtMTM1*, and *AtMTM2* were analyzed by qPCR. Mitochondrial oxidative-responsive gene *AOX1A* was used as a reference. Gene expression was normalized to *PP2A*. Data are mean ± SE of three independent repeats. Statistical significances (*P* < 0.05) among groups are indicated using different letters (Tukey’s HSD test).

To access the timing regulation of *AtMTM1* and *AtMTM2* for *AtMnSOD* activation, we treated seedlings with MV at different time periods (Figure 7). We examined AtMnSOD activity and protein levels in 14-day-old complete seedlings after 0.1, 1, 5, and 10 μM MV treatments for 24 h (Figure 7A). The AtMnSOD activity increased gradually with an increased dose of MV (Figure 7A, top), but the AtMnSOD protein level remained the same (Figure 7A, bottom). This result indicates the posttranscriptional regulation of AtMnSOD activity under MV stress.

We further elucidated *AtMnSOD*, *AtMTM1*, and *AtMTM2* gene expression profiles in 14-day-old complete seedlings after 5 μM MV treatment for 2, 4, 8, and 12 h by qPCR (Figure 7B). The rapidly upregulated *AtMnSOD* showed a trend similar to that of *AOX1A* in response to MV stress (Figure 7B, top), where *AOX1A*, a mitochondrial oxidative stress-responsive marker gene, was used as a reference. In particular, *AtMTM2* had an earlier and higher expression compared to *AtMTM1* in response to MV (Figure 7B, bottom). Overall, the *AtMnSOD*, *AtMTM1*, and *AtMTM2* genes are immediately upregulated in response to MV stress.

### Participation of *AtMTM1* and *AtMTM2* in Flowering-Time Control

**FIGURE 8:**
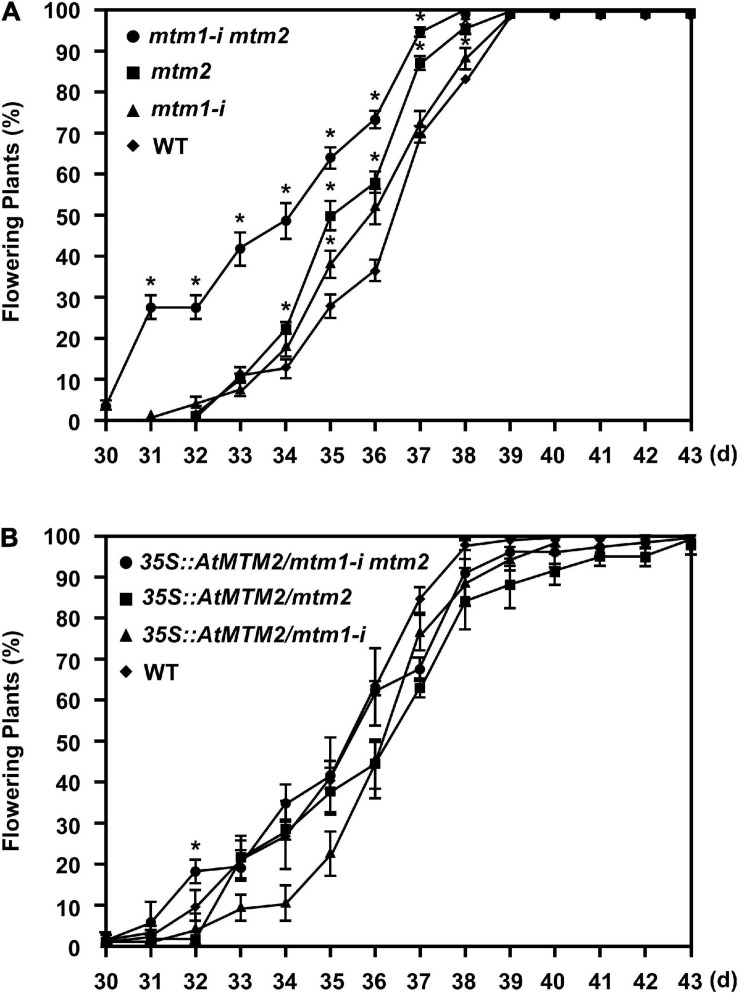
Flowering time analysis. **(A,B)** Percentages of flowering in WT, *mtm1-i*, *mtm2*, and *mtm1-i mtm2*-double mutants, as well as complementation lines with *AtMTM2* transgene in mutant background. Flowering time was scored when primary inflorescence reached 5 cm in length. Data are mean ± SE of three independent repeats (*n* = 60). *Significant at *P* < 0.05 (Student’s *t*-test).

Since the *mtm1-i mtm2*-double mutant indicated an early flowering phenotype (Supplementary Figure 5), we performed the flowering time analysis to elucidate the role of *AtMTM1* and *AtMTM2*. Both *mtm1-i* and *mtm2* single mutants displayed milder early flowering phenotypes compared with the WT, and the double mutant maintained the highest percentage of flowering plants (Figure 8A), indicating the synergistic effect of defective *AtMTM1* and *AtMTM2* at the flowering stage. The complementation lines of *35S:AtMTM2/mtm1-i*, *35S:AtMTM2/mtm2*, and *35S:AtMTM2/mtm1-i mtm2* showed flowering times similar to that of the WT (Figure 8B). These results indicate that *AtMTM1* and *AtMTM2* participate in flowering-time control.

### Phenotypic Complementation of Root Length in Mutants Through Mn Supply

**FIGURE 9:**
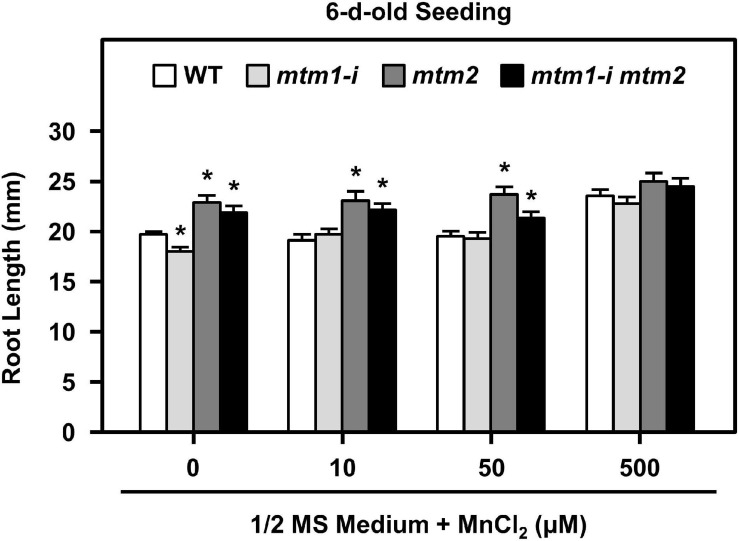
Root lengths of *mtm1-i*, *mtm2*, and *mtm1-i mtm2*-double mutants through Mn supply. Wild-type and three mutants were grown on 1/2 MS medium supplied without and with 10, 50, and 500 μM MnCl_2_ for 6 days. Data are mean ± SE of three independent repeats (*n* = 50). *Significant at *P* < 0.05 (Student’s *t*-test).

To delineate the defective metal regulation in transporter mutants, researchers have applied metal supplementation and examined phenotypic complementation (Schneider et al., 2016; Eisenhuta et al., 2018). We monitored the root lengths of *mtm1-i*, *mtm2*, and *mtm1-i mtm2*-double mutants on 1/2 MS plates containing 10, 50, and 500 μM MnCl_2_ (Figure 9). The 6-day-old *mtm1-i* seedlings showed a shorter root length compared with the WT without extra Mn supply, but *mtm2* and double mutants had longer root lengths. The 10, 50, and 500 μM Mn treatments restored the root length of *mtm1-i* on day 6, since *mtm1-i* and WT had similar root lengths through Mn supply. Moreover, the 500 μM Mn supply restored the root lengths of *mtm2* and the double mutant, since *mtm2* and *mtm1-i mtm2*-double mutants reached similar lengths compared with those in *mtm1-i* and WT. In summary, it is inferred that AtMTM1 and AtMTM2 are involved in root growth.

### Functional Role of AtMTM1 and AtMTM2 in Mn and Fe Homeostasis

**FIGURE 10:**
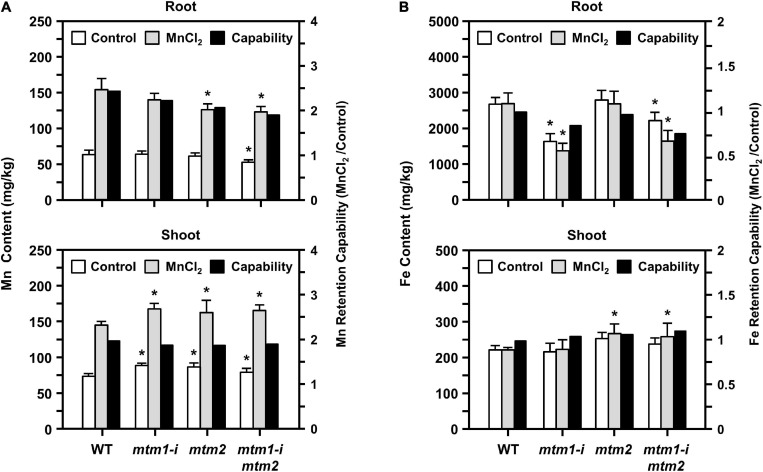
Manganese and Fe content in *mtm1-i*, *mtm2*, and *mtm1-i mtm*2-double mutants through Mn supply. **(A,B)** Metal contents (Mn and Fe) in root and shoot were measured by the ICP-OES; 14-day-old complete WT and mutant seedlings were incubated without (control) and with 100 μM MnCl_2_ for 24 h. Data are mean ± SE of three independent repeats. Metal retention capability represents the ratio of ion content with Mn supply (MnCl_2_) to that without Mn supply (control). *Significant at *P* < 0.05 (Student’s *t*-test).

To confirm the influence of defective AtMTM1 and AtMTM2 on Mn homeostasis, we maintained 14-day-old *mtm1-i*, *mtm2*, and *mtm1-i mtm2*-double mutants in 1/2 MS medium containing 100 μM MnCl_2_ for 24 h. The ICP-OES was performed to measure Mn and Fe ion contents in roots or shoots (Figure 10). The index of metal retention capability represents the ratio of the ion content with Mn supply to that without Mn supply.

All single and double mutants showed decreased Mn content and Mn retention capabilities in roots, especially in the *mtm2* and *mtm1-i mtm2*-double mutants (Figure 10A, top). This result indicated that the absorbance of Mn ions was restricted to the roots of these mutants. All mutants showed markedly increased Mn content in shoots under Mn treatment and displayed slightly decreased Mn retention capabilities in shoots (Figure 10A, bottom). These results imply that both AtMTM1 and AtMTM2 are involved in Mn homeostasis and that Mn ions may accumulate in the shoot.

The *mtm1* and double mutants showed decreased Fe content and Fe retention capabilities in the roots (Figure 10B, top). However, the *mtm2* and double mutants showed increased Fe content and retention capabilities in shoots under Mn treatment (Figure 10B, bottom). Fe metal retention capabilities were not consistent between roots and shoots, indicating that AtMTM1 and AtMTM2 exhibit divergence in Fe homeostasis.

### Activation of Chloroplast-Destined AtMnSOD

**FIGURE 11:**
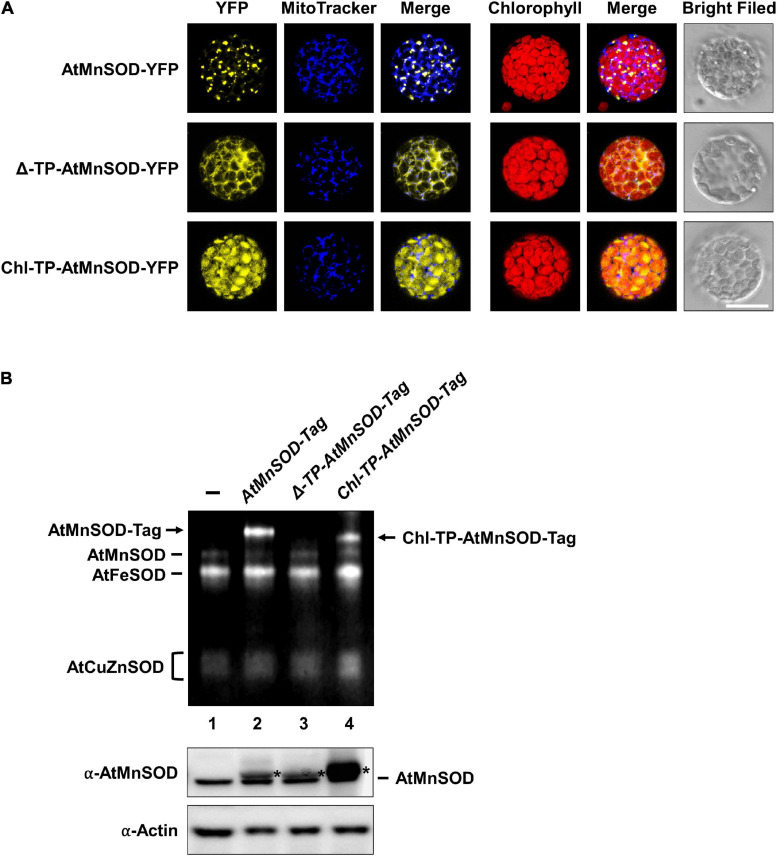
Subcellular localization and SOD activity assay of cytosol-destined and chloroplast-destined AtMnSOD. **(A)** Transfection of *AtMnSOD-YFP*, Δ*-TP-AtMnSOD-YFP*, and *Chl-TP-AtMnSOD-YFP* transgenes in WT protoplasts and YFP signals corresponded to the localization of mitochondrial, cytosol, and chloroplast, respectively. Bar = 20 μm. **(B)** Transient expressions without (–) and with *AtMnSOD-Tag*, Δ*-TP-AtMnSOD-Tag*, and *Chl-TP-AtMnSOD-Tag* transgenes in WT protoplasts were analyzed using the in-gel SOD activity assay **(top)** and western blotting with α-AtMnSOD and α-actin antibodies **(bottom)**. Actin was a loading control. *Represents the exogenous-expressed AtMnSOD.

It has been shown that the Mn insertion is linked to the ySOD2 importing process via yMTM1 in mitochondria (Luk et al., 2005), and the chloroplastic-localized MnSOD exists in some algae. To analyze whether AtMnSOD can be activated in the cytosol and chloroplasts, we constructed cytosol-destined AtMnSOD (Δ-TP-AtMnSOD) and chloroplast-destined AtMnSOD (Chl-TP-AtMnSOD) (Supplementary Figure 1A), which were then fused with YFP to confirm their localization by transient expression in protoplasts (Figure 11A). Confocal microscopy revealed that the localization of AtMnSOD-YFP, Δ-TP-AtMnSOD-YFP, and Chl-TP-AtMnSOD-YFP was consistent with their destinations in the mitochondria, cytosol, and chloroplasts, respectively.

AtMnSOD and the modified AtMnSOD were also fused with a tag (Supplementary Figure 1B), and their activities could be distinguished from the endogenous AtMnSOD by using the in-gel SOD activity assay (Figure 11B). Exogenously expressed AtMnSOD-Tag and Chl-TP-AtMnSOD-Tag were activated (Figure 11B, lanes 2 and 4), but not the Δ-TP-AtMnSOD-Tag (Figure 11B, lane 3). This result agrees with the importance of the mitochondrial MTM for MnSOD activation (Luk et al., 2005), and an evolutionarily conserved activation mechanism of MnSOD still exists in *Arabidopsis* chloroplasts.

## Discussion

Mitochondrial carrier family proteins are localized in the mitochondrial inner membrane, and they mediate the transport of inorganic ions, cofactors, metabolites, and nucleotides from the cytosol into the mitochondrial matrix (Haferkamp and Schmitz-Esser, 2012). The *yMTM1* transgene complements ySOD2 activity in *ymtm1*Δ cells (Luk and Culotta, 2001; Luk et al., 2003, 2005). This study revealed that the mitochondrial carrier family proteins AtMTM1 and AtMTM2 are homologs of yMTM1 by sequence comparison and transformation assay (Figures 1, 2). The transformation of *AtMTM1-* or *AtMTM2*-enhanced ySOD2 activity reflected the maximum capacity in yeast mutant cells; however, co-transformation of *AtMTM1* and *AtMTM2* resulted in similar ySOD2 activity as the WT. There may be a competition effect of AtMTM1 and AtMTM2, or unknown cellular factors were involved during the co-transformation. We demonstrated that AtMTM1 and AtMTM2 are necessary for AtMnSOD activation by using transient expression assay (Figure 3). We confirmed that AtMTM1 and AtMTM2 interact with AtMnSOD in mitochondria by using the BiFC assay (Figure 4), which agrees with the proteomic evidence of mitochondrial AtMTM1 and AtMTM2 (Senkler et al., 2017).

Analysis of the expression of *Arabidopsis* mitochondrial carrier family proteins has revealed that carriers usually have distinct specificities during plant growth and abiotic stress (Maia et al., 1998; Watanabe et al., 1999; Catoni et al., 2003; Hoyos et al., 2003). In this study, the gene expression levels of *AtMTM1*, *AtMTM2*, and *AtMnSOD* were measured relative to *AtMTM1* level in the root or in the control, and thus, we can compare the relative ratio in tissues and in metal stresses. *AtMTM1* and *AtMTM2* were ubiquitously expressed in different tissues and were responsible for metal stress in the 14-day-old complete seedlings (Figure 5). However, the substrate specificity or the metal ion affinity of AtMTM1 and AtMTM2 remains to be clarified. We used MV as a superoxide generator and emphasized the complementary effect of *AtMTM2* transgene in the mutants. The complementation lines of *35S:AtMTM2/mtm1-i* and *35S:AtMTM2/mtm2* partially restored the root lengths to the WT in response to MV stress in 8- to 10-day-old seedlings (Figure 6). The MV sensitivities of the root lengths in these complementation lines may be restricted to different degrees based on their background or the germination process, and it is worthwhile to use several independent complementation lines with different *AtMTM2* alleles or monitor the root length from germination to rule out these possibilities. The study further revealed an increase in AtMnSOD activity under MV stress, with *AtMTM2* expression being dominant compared with *AtMTM1* in the 14-day-old complete WT seedlings (Figure 7); thus, it is worthwhile to track the root lengths for a longer time in these complementation lines. WT seedling remained bright green rosette leaves after MV stress, and it is worthwhile to detect *AtMTM1*, *AtMTM2*, and *AtMnSOD* gene expressions at 24 h and monitor the AtMnSOD activity in *mtm1-i*, *mtm2*, and double mutants under MV stress to support the posttranslational regulation of AtMnSOD. Taken together, we assume that *AtMTM1* and *AtMTM2* have different sensitivities in response to metal and MV stress and that both AtMTM1 and AtMTM2 carriers are involved in the posttranscriptional regulation of AtMnSOD. Notably, the complementation lines of *35S:AtMTM2/mtm1-i*, *35S:AtMTM2/mtm2*, and *35S:AtMTM2/mtm1-i mtm2* reverted from the early flowering phenotype to the WT (Figure 8), which reflects the necessity of *AtMTM1* and *AtMTM2* for flowering-time control.

Yeast yMTM1 facilitates the insertion of the Mn cofactor into ySOD2, but *ymtm1*Δ retains normal Mn levels in the mitochondria (Luk et al., 2003). The loss of the Mn transporter SMF2 decreases ySOD2 activity and Mn levels in yeast (Luk and Culotta, 2001; Luk et al., 2005). The disruption of vacuole-localized Mn transporters of NRAMP3 and NRAMP4 decreases the amount of photosystem II, but does not affect MnSOD activity in *Arabidopsis* (Allen et al., 2007; Lanquar et al., 2010). Mn-deficient *Arabidopsis* has abnormal root lengths and altered Fe homeostasis (Yang et al., 2008; Rodríguez-Celma et al., 2016). In our study, the defective Mn carriers of AtMTM1 and AtMTM2 decreased AtMnSOD activity in the *mtm1-i mtm2*-double mutant. The Mn supply can restore the abnormal root lengths of *mtm1-i*, *mtm2*, and *mtm1-i mtm2*-double mutants to the WT (Figure 9); the strengthened uptake of Mn via AtMTM1 and AtMTM2 can compensate the altered root length phenotype on day 6. It is worthwhile to monitor the root length from the germination, since *mtm1-i* and *mtm2* showed divergent root lengths on day 6 and reached similar lengths as that in WT on day 8 to day 10 under control conditions. We used the same seedling stage and the same condition in the ICP-OES assay and the gene expression assay under Mn stress, which showed upregulated gene expressions in WT. The decreased Mn content in the 14-day-old *mtm1-i* and *mtm2* roots also indicates that AtMTM1 and AtMTM2 are associated with Mn homeostasis. The decreased Mn retention capability was more obvious in the *mtm1-i mtm2*-double mutant, implying that both AtMTM1 and AtMTM2 have a higher affinity for Mn (Figure 10). In contrast, *ymtm1*Δ mutant had decreased ySOD2 activity, with increased ISU protein and higher Fe content. ySOD2 inactivation has also been observed in the Fe–S cluster biogenesis mutants of *atm1*Δ, *grx5*Δ, and *ssq1*Δ (Luk et al., 2003; Naranuntarat et al., 2009). The decreased Fe content in the roots of *mtm1-i* and the double mutant in our study implies that AtMTM1 has a higher affinity for Fe.

In addition to land plants, MnSOD is present in the thylakoids of some prokaryotic and eukaryotic algae. MnSOD participates in photosynthetic water oxidation and scavenges superoxide in the chloroplasts of algae, which is influenced by the environment with fluctuating light and temperature (Kanematsu and Asada, 1979; Okada et al., 1979; Regelsberger et al., 2002). However, this function in the chloroplasts was taken over by FeSOD in *Arabidopsis*. In our study, we generated modified AtMnSOD constructs and noticed that chloroplast-destined AtMnSOD can be activated in *Arabidopsis* protoplasts (Figure 11). The factors involved in chloroplast-destined AtMnSOD activation may include envelop-localized or thylakoid-localized Mn transporters of NRAMP3, NRAMP4, CMT1, PAM71/CCHA1, Mn cluster-related factors, or released Mn from photosystem II (Schneider et al., 2016; Wang et al., 2016). Any chloroplast Mn transporter that has a structure similar to that of AtMTM1 or AtMTM2 may insert Mn into modified AtMnSOD polypeptides. Likewise, modified AtMnSOD may have a structure similar to that of certain chloroplast proteins and accept Mn via its Mn transporter. Thus, the activity of chloroplast-destined AtMnSOD highlighted an evolutionarily conserved mechanism that remains in *Arabidopsis* chloroplasts, as the chloroplastic-localized MnSOD in algae.

## Conclusion

This study demonstrates that AtMTM1 and AtMTM2 interact with AtMnSOD, respond to oxidative stress, regulate the root growth, participate in flowering-time control, and involve in Mn homeostasis. We elucidated the gene expression profiles of *AtMnSOD*, *AtMTM1*, and *AtMTM2* during development. The indispensability of mitochondrial carrier proteins AtMTM1 and AtMTM2 for AtMnSOD enzyme activation was well established by this study. We also modified AtMnSOD and found that chloroplastic factors for chloroplast-destined AtMnSOD activation still exist. Overall, this study reveals the physiological roles of AtMTM1 and AtMTM2 in MnSOD activation and links the Mn uptake via AtMTM1 and AtMTM2 to the MnSOD importing process in mitochondria.

## Data Availability Statement

The original contributions presented in the study are included in the article/Supplementary Material, further inquiries can be directed to the corresponding author.

## Author Contributions

All authors listed have made a substantial, direct and intellectual contribution to the work, and approved it for publication.

## Conflict of Interest

The authors declare that the research was conducted in the absence of any commercial or financial relationships that could be construed as a potential conflict of interest.

## Publisher’s Note

All claims expressed in this article are solely those of the authors and do not necessarily represent those of their affiliated organizations, or those of the publisher, the editors and the reviewers. Any product that may be evaluated in this article, or claim that may be made by its manufacturer, is not guaranteed or endorsed by the publisher.

## Footnotes

1
http://www-sequence.stanford.edu/group/yeast_deletion_project

2
http://primer3.ut.ee

3
http://wmd3.weigelworld.org/cgi-bin/webapp.cgi

4
http://blast.ncbi.nlm.nih.gov/Blast.cgi

## Supplementary Material

Supplementary Table 1 Primers for genotyping, cloning, RT−PCR, and qPCR, as well as accession numbers of genes.

Supplementary Figure 1 Constructs of modified AtMnSOD.

Supplementary Figure 2 Characterization of *mtm1-1* and *mtm1-2* T-DNA insertion mutants.

Supplementary Figure 3 Characterization of *mtm1-i* mutant.

Supplementary Figure 4 Characterization of *mtm2-1*, *mtm1-2*, and *mtm1-3* T-DNA insertion mutants.

Supplementary Figure 5 Characterization of *mtm1-i mtm2*-double mutant.

Supplementary Figure 6 Control experiments in BiFC.

Supplementary Figure 7 Histochemical analysis of *AtMTM2*-promoter:*GUS* plants.
