# Supplementary material for: Significance of AtMTM1 and AtMTM2 for Mitochondrial MnSOD Activation in Arabidopsis
Source: Front Plant Sci. 2021 Aug 6;12:690064. doi: 10.3389/fpls.2021.690064 (PMC8382117; doi:10.3389/fpls.2021.690064)
Supplement: Supplementary Table 1 — Primers for genotyping, cloning, RT−PCR, and qPCR, as well as accession numbers of genes. [file Data_Sheet_1.pdf]

Supplementary Table S1. Primers for cloning, qPCR, genotyping and RT-PCR, as well as accession numbers of genes.

**Purpose**	**Sequence (5'→ 3')**	**Primer Set**
Expression of *AtMTM1* in yeast	TCTGAATTCATGGTTGAGGCAGAG	AtMTM1-Fw-EcoRI
	TCTGAATTCTGATGAAGCATGGCGATGCA	AtMTM1-Rv-EcoRI
Expression of *yMTM1* in yeast	CAGAATTCTCACTGATGCAATTTGTTTC	yMTM1-Fw-EcoRI
	CAGAATTCATGAGTGATCGCAATACAAG	yMTM1-Rv-EcoRI
Transient expression of *AtMTM1-YFP* in protoplasts	CAAAGCTTATGGTTGAGGCAGAGCGC	AtMTM1-YFP-Fw-HindIII
	CAGAATTCTGATGAAGCATGGCGATGCAG	AtMTM1-YFP-Rv-EcoRI
Construction of *AtMTM1* for BiFC	ATGGTTGAGGCAGAGCGCG	AtMTM1-Gateway-Fw
	TGATGAAGCATGGCGATGCAGA	AtMTM1-Gateway-Rv
qPCR for *AtMTM1* expression	AGAGGACTGTTCATGGGAATGG	AtMTM1-qPCR-Fw
	ATACTTGACCACTTCGTAGAAGGAAAC	AtMTM1-qPCR-Rv
Genotyping of *mtm1-1*	GCAGAGCGCGCGGAGAATA	AtMTM1-LP1
	CGACATAAACCGGATTAAACATTA	AtMTM1-RP1
Genotyping of *mtm1-2*	ACTCAAGTCTCCAGTGTTCCT	AtMTM1-LP2
	AATAGAAAGCTCCTACCTTGA	AtMTM1-RP2
Construction for *mtm1-i*	CAGAGCTCTGCACAACTATCGTGGTCTCT	AtMTM1-Fw1-SacI
	CAGAGCTCTTGACCACTTCGTAGAAGGAA	AtMTM1-Rv1-SacI
	CAGGATCCTGCACAACTATCGTGGTCTCT	AtMTM1-Fw2-BamHI
	CATCTAGATTGACCACTTCGTAGAAGGAA	AtMTM1-Rv2-XbaI
Expression of *AtMTM2* in yeast	TCTCTCGAGATGGTGGAAGAAGCACAATTG	AtMTM2-Fw-XhoI
	CACTCGAGTTACTGTTGGTGGAAGTTGT	AtMTM2-Rv-XhoI
Transient expression of *AtMTM2-YFP* in protoplasts	CAAAGCTTATGGTGGAAGAAGCACAATTG	AtMTM2-YFP-Fw-HindIII
	CAAAGCTTCTGTTGGTGGAAGTTGTGCAG	AtMTM2-YFP-Rv-HindIII
Construction of *AtMTM2* for BiFC and *35S::AtMTM2*	ATGGTGGAAGAAGCACAAT	AtMTM2-Gateway-Fw
	CTGTTGGTGGAAGTTGTGCAGT	AtMTM2-Gateway-Rv
Construction of *AtMTM2*-promoter*::GUS*	CAGAATTCAGAAATAAAAAAAAAGGACCATT	AtMTM2-Pro-Fw-EcoI
	CACCATGGCAGGAAAAAACAAAACTCGAT	AtMTM2-Pro-Rv-NcoI
qPCR for *AtMTM2* expression	GAGCCATGACAATGACCACAAG	AtMTM2-qPCR-Fw
	CTCTAGCCCCTGCTCCACTAAAC	AtMTM2-qPCR-Rv

Genotyping of *mtm2-1*	CTTGGCTTGTATCTCTTGTTA	AtMTM2-LP
	CTGTTGGTGGAAGTTGTGCAGT	AtMTM2-RP
Genotyping of *mtm2-2* and *-3*	ATGGTGGAAGAAGCACAAT	AtMTM2-LP1
	CTACAGATGGTCCTGCTCGTC	AtMTM2-RP1
RT-PCR for *AtMTM2* expression	GGCTCAAATAATGGATATAGGATGTT	AtMTM2-RT-Fw
	TACTTGACCACTTCGTAAAACGATAC	AtMTM2-RT-Rv
Expression of *AtMSD1* in yeast	TCTGAATTCATGGCGATTCGTTGTGTAGCGAG	AtMSD1-Fw-EcoRI
	TCTGAATTCTCAGTTGTTTTCCTTCTCATAAACCTC	AtMSD1-Rv-EcoRI
Construction of *AtMSD1* for BiFC	ATGGCGATTCGTTGTGTAGCGAG	AtMSD1-Gateway-Fw
	GTTGTTTTCCTTCTCATAAACCTC	AtMSD1-Gateway-Rv
qPCR for *AtMSD1* expression	TGCCATTGACGCTCACTTTG	AtMSD1-qPCR-Fw
	TTGTCTAGTCCGAGCCACAC	AtMSD1-qPCR-Rv
qPCR for Arabidopsis *AOX1A* expression	GAATGTTCCTGCTCCGGCTAT	AOX1A-qPCR-Fw
	TCAGCACGAACAACCATCACA	AOX1A-qPCR-Rv
T-DNA left border	ATTTTGCCGATTTCGGAAC	BP (pROK2 LBb 1.3)
Internal control of RT-PCR	GATCTTTGCCGGAAAACAATTGGAGGATGGT	UBQ10-Fw
	CGACTTGTCATTAGAAAGAAAGAGATAACAGG	UBQ10-Rv
Internal control of qPCR	CCTGCGGTAATAACTGCATCT	PP2A-Fw
	CTTCACTTAGCTCCACCAAGCA	PP2A-Rv
Construction of chloroplast transit peptide of Arabidopsis CSD2	CAGAATTCATGGCTGCCACCAACACAATCCT	Chl-TP-CSD2-Fw-EcoRI
	CACCATGGAAGCACTGCAACAGCCTTCT	Chl-TP-CSD2-Rv-NcoI

Sequence data in this article can be found in the TAIR and Saccharomyces genome database (SGD) under the following accession numbers: *AOX1A* (At3g22370), *CSD2* (At2g28190), *AtMSD1* (At3g10920), *AtMTM1* (At4g27940), *AtMTM2* (At2g46320), *PP2A* (At1g13320), *UBQ10* (At4g05320), and *yMTM1* (YGR257C).

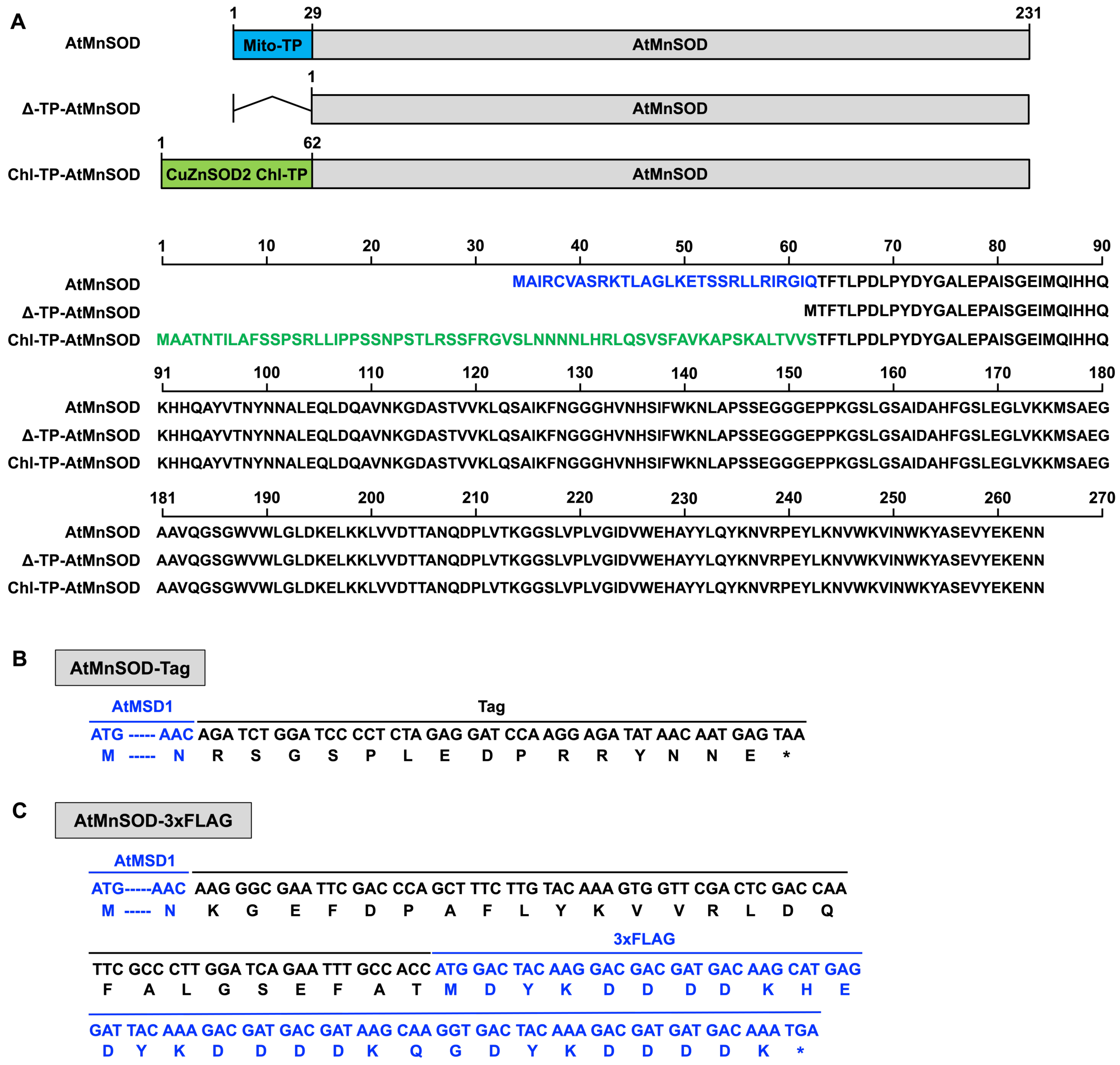

Supplementary Figure S1. Constructs of modified AtMnSOD. (A) Δ-TP-AtMnSOD was generated by deleting the first 29 amino acids of mitochondrial transit peptide (Mito-TP) of AtMnSOD for its expression in the cytosol. Chl-TP-AtMnSOD was generated by fusing Δ-TP-AtMnSOD with the first 62 amino acids of chloroplast transit peptide of Arabidopsis CuZnSOD2 (Chl-TP) for delivering AtMnSOD to the chloroplastic stroma. **(B)** AtMnSOD-Tag was generated by fusing a tag of 15 amino acids derived from the vector to the C-terminal end of AtMnSOD. **(C)** AtMnSOD-3xFLAG was generated by fusing a 3xFLAG to the C-terminal end of AtMnSOD.

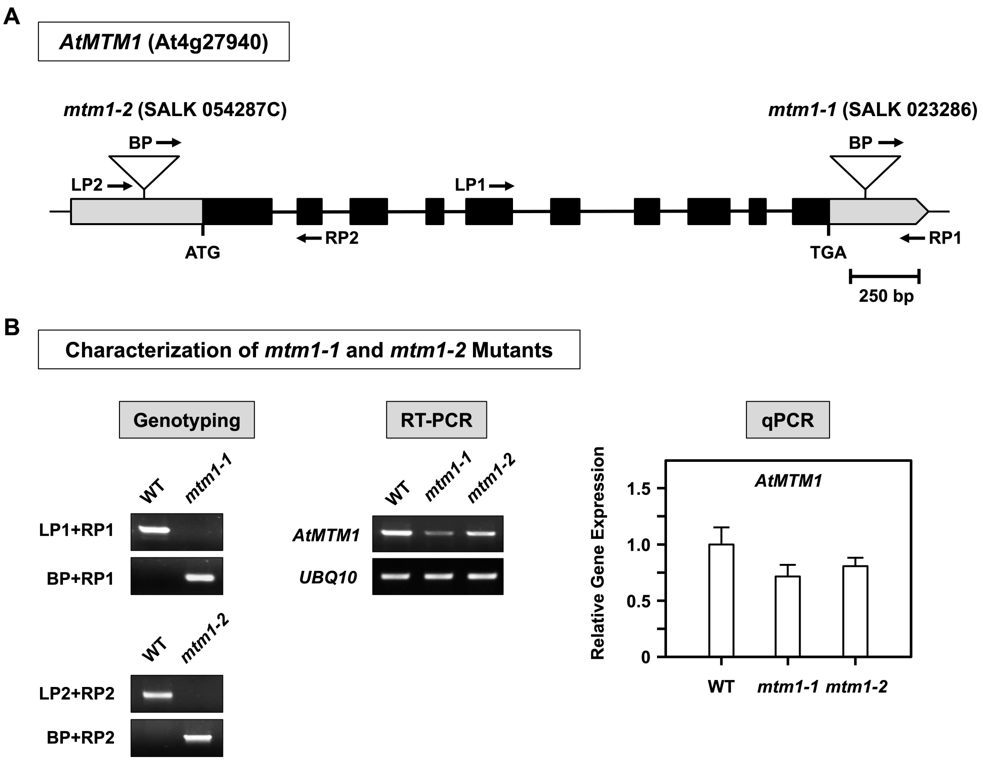

Supplementary Figure S2. Characterization of T-DNA insertion mutants of *mtm1-1* and *mtm1-2*. (A) Schematic map of *AtMTM1* gene and T-DNA insertion sites. The grey box, black box and solid line indicate UTR region, exon and intron respectively. T-DNA insertion sites are indicated as triangles. Primers for genotyping are indicated as arrows. ATG and TGA are shown as initiation and stop codons respectively. **(B)** Genotyping by PCR was analyzed with DNA flanking sequence primers (LP and RP) and T-DNA border primer (BP). RT-PCR and qPCR involved *AtMTM1* gene-specific primers. *AtMTM1* gene expression in mutants was measured relative to that in WT. *Ubiquitin10* (*UBQ10*) and *PP2*A were internal controls of RT-PCR and qPCR, respectively.

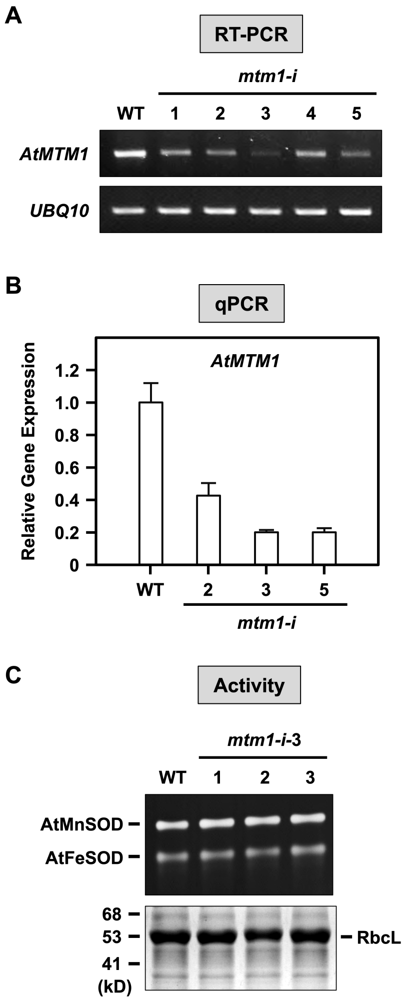

Supplementary Figure S3. Characterization of miRNA-mediated *AtMTM1* suppression (*mtm1-i*) mutants. (A) Five independent *mtm1-i* lines in T4 generation were analyzed by RT-PCR. *UBQ10* was an internal control. **(B)** Three down-regulated *mtm1-i* lines were analyzed by qPCR. *AtMTM1* gene expression in mutants was measured relative to that in WT. *PP2A* was an internal control. **(C)** In-gel SOD activity assay of *mtm1-i*-3 seedlings **(top)** and Coomassie blue staining gel **(bottom)** were conducted. The ribulose bisphosphate carboxylase large subunit (RbcL) was a loading control.

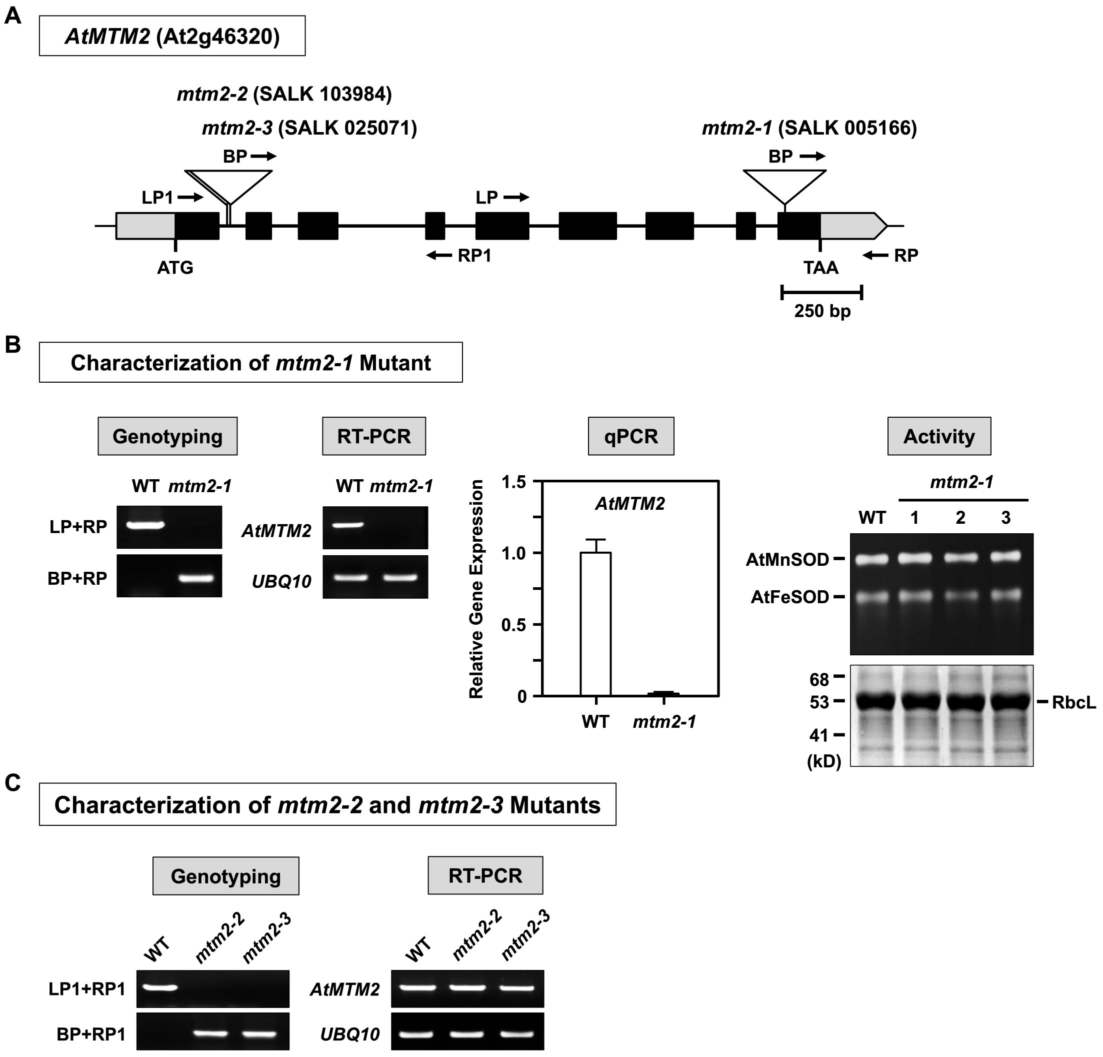

Supplementary Figure S4. Characterization of T-DNA insertion mutants of *mtm2-1*, *mtm2-2*, and *mtm2-3*. **(A)** Schematic map of *AtMTM2* gene and T-DNA insertion sites. **(B and C)** Genotyping by PCR was performed with DNA flanking sequence primers (LP and RP) and T-DNA border primer (BP). RT-PCR and qPCR involved *AtMTM2* gene-specific primers. *AtMTM2* gene expression in the mutant was measured relative to that in WT. *UBQ10* and *PP2A* were internal controls of RT-PCR and qPCR, respectively. In-gel SOD activity assay of *mtm2-1* seedlings **(top)** and Coomassie blue staining gel **(bottom)** were conducted. RbcL was a loading control.

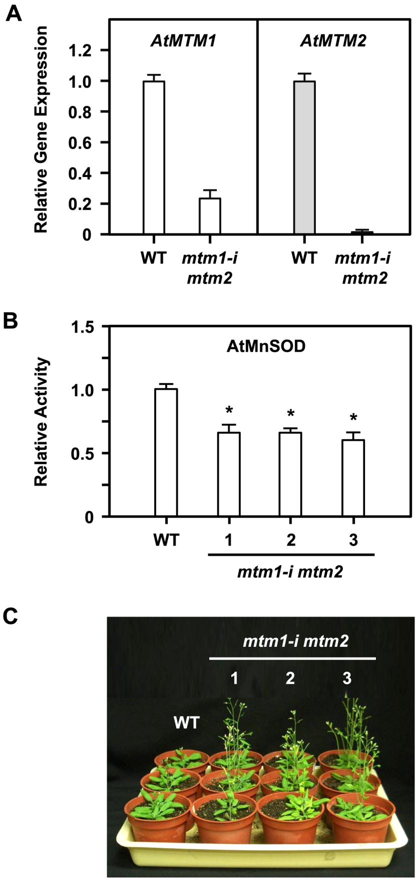

Supplementary Figure S5. Characterization of *mtm1-i mtm2*-double mutants. (A) The expression levels of *AtMTM1* and *AtMTM2* in double mutants were analyzed by qPCR. The expression levels were measured relative to that in WT. *PP2A* was an internal control. **(B)** Relative AtMnSOD activity of three independent double mutant lines. AtMnSOD activity in mutants was measured relative to that in WT. Data are mean ± SE of three independent repeats. *, significant at $P < 0.05$ (Student's t test). **(C)** The early-flowering phenotype of one-month-old double mutants.

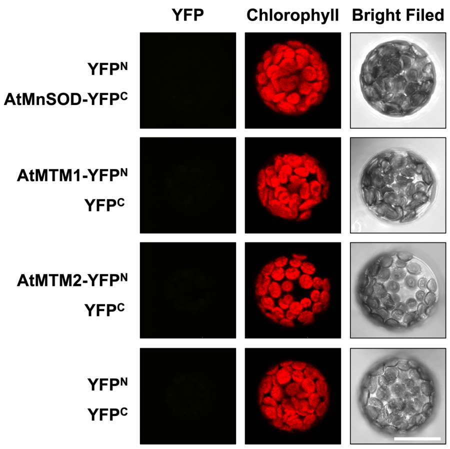

Supplementary Figure S6. Control experiments in BiFC assay. The YFP signals were not detected in the co-transfections of YFP^N and AtMSD1-YFP^C, AtMTM1-YFP^N and YFP^C, AtMTM2-YFP^N and YFP^C, as well as YFP^N and YFP^C. Bar = 20 μm.

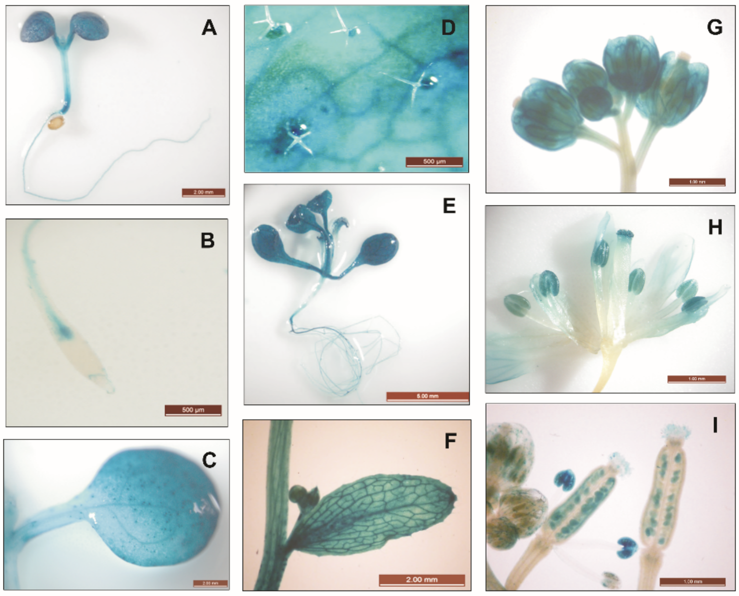

Supplementary Figure S7. Histochemical analysis of *AtMTM2*-promoter::*GUS* plants. GUS staining in **(A)** 5-d-old seedling, **(B)** root, **(C)** cotyledon, **(D)** trichomes and vascular bundles, **(E)** 15-d-old seedling, **(F)** stem and cauline leaf, **(G)** flower bud cluster, **(H)** flower, and **(I)** young siliques. Bars in A, C, F = 2 mm; B, D = 500 µm; E = 5 mm; G, H, I = 1 mm.
